# Penile Paraffinoma

**DOI:** 10.1155/2012/202840

**Published:** 2012-09-17

**Authors:** Necmi Bayraktar, İsmet Başar

**Affiliations:** ^1^Akcicek State Hospital, Kyrenia, North Cyprus, Turkey; ^2^Nalbantoğlu State Hospital, Nicosia, North Cyprus, Turkey

## Abstract

Penile paraffinoma is an uncommon entity produced by penile paraffin injections for the purpose of penile enlargement by a nonmedical person. Although it is not a current method of penile enlargement procedures, in our opinion dermatologists and urology specialist should be have knowledge of this entity about diagnosis and management. It will be an aim to share our experiences and views in this paper.

## 1. Introduction 

 Penile paraffinoma, or as named in old terms sclerosing lipogranuloma of male genitalia, is an uncommon entity produced by penile paraffin injections for the purpose of penile enlargement [1, 2]. Generally, penile subcutaneous and glandular paraffin injections for penile augmentation are performed by a nonmedical person, under unacceptable conditions. It usually occurs months to years after the injections. Unfortunately the injections are generally repeated a number of times in order to reach the desired enlargement and shape, which in turn causes the early complications such as infection, allergic reactions, paraphimosis (circumcised or uncircumcised), severe pain, or tenderness and inflammatory reactions.

 In 1899, Robert Gersuny who is an Austrian surgeon from Vienna injected mineral oil (Vaseline) to substitute the absence of testicles in a patient who had undergone bilateral orchiectomy for tuberculosis epididymitis [1, 3]. The immediate success of the operation encouraged him to use Vaseline as filling material for soft tissue defects. Human body lacks the enzymes to metabolize interstitial exogenous oils [4]. So, a foreign body reaction will inevitably cause a subcutaneous paraffin deposition. Complications of the injection of these oil substances are well known and had been reported in 1906 in two patients who had received paraffin injections for facial wrinkles and developed defacing subcutaneous nodules. The principle of the technique was the injection of a product that becomes semiliquid by heating, but it solidifies when it gets colder. It remains stable in the human body. It was used for the cure of palatal defects, as well as urinary fistulae and hernia repairs but was mainly used for cosmetic purposes: for the filling of wrinkles of face, cheeks, and frontal areas and for breast augmentation as well as penile reconstruction. Although serious complications had been reported, it remained popular for the first 20 years of the 20th century. Unfortunately, even with initial good results, secondary or late severe complications appeared due to the deposition of paraffin. There was formation of nodules called lipogranulomas, which were very difficult to remove. Despite the severe destructive outcomes, this procedure is still popular in some parts of the world, such as Asia and Eastern European Countries [5–7].

## 2. General Clinical Characteristics

- Amorphous changes and swelling on penile skin.
- In the vast majority of the cases the purpose of paraffin injections is penile enlargement.
- Penile pain or discomfort with erection.
- Decreased rigidity of the penis because of pain.
- History of multiple mineral oil injections by a non-medical person.
- A rapid recurrence in case of incomplete excision.

## 3. Case Report

 We report two cases of 19- and 22-year old circumcised men who presented with multiple, irregular, nodular, and tender penile masses, amorphous skin changes, and painful erections. The four cardinal signs of inflammation, (*color, dolor, tumor, and rubor*) were present on physical examination of both patients. There was no ulceration, strangulation, or inguinal lymph node involvement. They had no systemic diseases previously. Penile injections had been performed 5-6 days before presentation, by the same untrained non-medical person, whose main job was car cleaning. He used liquid paraffin for the injections. 

## 4. Findings

 There were no abnormalities related to the laboratory findings including complete blood count, blood chemistry, and urine analysis. Radiological studies, which included chest X-ray and abdominal ultrasonography, were also normal.

## 5. Treatment 

 Although it was suggested in the literature that all masses should be excised together with the skin, for definitive treatment [3, 7–9], because of the severe acute inflammatory reactions, our initial treatment was confined to medical measures for the first two weeks with second-generation cephalosporin, nonsteroidal anti-inflammatory drugs (NSAID), and antihistaminic medications. When local physical reactions were resolved all masses were excised under general anesthesia without the need for a skin graft or a flap. Unfortunately one patient developed recurrent lesions 8 weeks after surgery, probably due to incomplete resection. Excisions of recurrent lesions were performed. Both patients were followed periodically once every three months for monitoring cosmetic results and sexual function. During the follow-up period of 2 years there was no evidence of recurrent lesions or sexual dysfunction and there was also no need for further medications.

## 6. Histological Evaluation

 Pathologically, granulomatous reaction with nodular pattern was shown on all specimens without any evidence of malignancy. 

## 7. Discussion 

 Although it is a rare entity, urologists and dermatologists should be aware of paraffinomas. Differential diagnosis of other reasons of subcutaneous nodules is essential [5]. Detailed patient history is the most important evidence for the diagnosis of paraffinomas, probably more helpful than pathological examination. Paraffin, Vaseline, or mineral oils are the most common materials used for injection. Almost always, these procedures are recommended and performed by an untrained non-medical person. Complete removal of the lesion should be considered as the only effective and proper treatment. No spontaneous regressions of paraffinomas have been reported. Many uninformed patients are candidates to accept the oil injection procedure because of the low procedural costs and because of the unreal misdirection and reward of penile augmentation and high sexual performance for them and for their partners without side effects. At this point public information is important about penile augmentation and healthy sexual life [3, 7, 10]. Furthermore, alternative minimal invasive, cost-effective, reliable, and safe penile augmentation procedures are necessary to replace illegal unsafe procedures and to avoid the misuse of herbal medications.
